# Preoperative therapy with angiotensin-converting enzyme inhibitors in cardiac surgery patients: is there any impact on postoperative renal function?

**DOI:** 10.1186/cc13378

**Published:** 2014-03-17

**Authors:** F Ampatzidou, M Sileli, K Diplaris, C Koutsogiannidis, T Karaiskos, G Drossos

**Affiliations:** 1 General Hospital 'G. Papanikolaou, Thessaloniki, Greece

## Introduction

Preoperative therapy with angiotensin-converting enzyme inhibitors (ACEI) is common in patients undergoing cardiac surgery. The aim of this study was to evaluate the still-debated impact of preoperative use of ACEI on postoperative renal function in cardiac surgery patients [1],[2].

## Methods

A total of 624 consecutive patients, who underwent cardiac surgery from July 2012 to October 2013, were evaluated. Data were prospectively collected in our clinic's electronic database and were retrospectively analyzed as to preoperative ACEI therapy. The chi- square test was used for correlations. Endpoints of the study were the development of postoperative acute kidney injury (AKI) and the difference between hospital admission and discharge glomerular filtration rate (GFR). The AKI definition was based on modified RIFLE classification. GFR values were estimated by the MDRD formula.

## Results

A total of 354 patients (56.7%) were treated with ACEI preoperatively. Overall, 95 patients (15.3%) developed postoperative AKI. Preoperative use of ACEI was not associated with the development of postoperative AKI (*P *= 0.981). Mean GFR values on admission day were 65.23 ± 16.89 for ACEI users, and 65.06 ± 19.62 for the rest of the cohort. Mean GFR values on discharge day were 66.77 ± 21.25 and 67.35 ± 25.64 respectively. The difference in GFR at the time of hospital admission and on discharge day had no statistical difference, *P *= 0.511 (Table 1). Overall, 25 patients (4%) needed dialysis.

**Table 1:** AKI and GFR difference

	ACEI users (*n *= 354)	ACEI nonusers (*n *= 270)	*P *value
AKI	54 (15.3%)	41 (15.2%)	0.981
GFR difference (mean)	+ 1.43	+2.3	0.511

## Conclusion

The impact of preoperative ACEI treatment on postoperative renal function after cardiac surgery is still debated. We found no association, neither protective nor harmful, between preoperative ACEI therapy and renal function impairment.
